# Global Container Port Network Linkages and Topology in 2021

**DOI:** 10.3390/s22155889

**Published:** 2022-08-07

**Authors:** Lu Kang, Wenzhou Wu, Hao Yu, Fenzhen Su

**Affiliations:** 1 State Key Laboratory of Resources and Environmental Information System, Institute of Geographical Sciences and Natural Resources Research, Chinese Academy of Sciences, Beijing 100101, China; 2 College of Resources and Environment, University of Chinese Academy of Sciences, Beijing 100049, China; 3 Collaborative Innovation Center of South China Sea Studies, Nanjing University, Nanjing 210093, China

**Keywords:** global liner shipping network, port accessibility, Space-L, complex network, maritime transport, shipping community detection

## Abstract

The maritime transport of containers between ports accounts for the bulk of global trade by weight and value. Transport impedance among ports through transit times and port infrastructures can, however, impact accessibility, trade performance, and the attractiveness of ports. Assessments of the transit routes between ports based on performance and attractiveness criteria can provide a topological liner shipping network that quantifies the performance profile of ports. Here, we constructed a directed global liner shipping network (GLSN) of the top six liner shipping companies between the ports of Africa, Asia, North/South America, Europe, and Oceania. Network linkages and community groupings were quantified through a container port accessibility evaluation model, which quantified the performance of the port using betweenness centrality, the transport impedance among ports with the transit time, and the performance of ports using the Port Liner Shipping Connectivity Index. The in-degree and out-degree of the GLSN conformed to the power-law distribution, respectively, and their R-square fitting accuracy was greater than 0.96. The community partition illustrated an obvious consistence with the actual trading flow. The accessibility evaluation result showed that the ports in Asia and Europe had a higher accessibility than those of other regions. Most of the top 30 ports with the highest accessibility are Asian (17) and European (10) ports. Singapore, Port Klang, and Rotterdam have the highest accessibility. Our research may be helpful for further studies such as species invasion and the planning of ports.

## 1. Introduction

Over 80% of international trade volume, accounting for 70% of its trade value, is carried by ships and handled by seaports around the world [1]. Maritime transport is regarded as the backbone of global trade and the lifeblood of the global economy. The operational performance of ports and the links between ports together form the maritime transport network, which has an important influence on global maritime trade [2]. Thus, research on maritime transport networks as well as port connectivity and accessibility has received increasing attention in recent years [3,4].

Traditional shipping studies relied upon indicators such as the GDP or freight index to analyze trade volume and the value between countries [5,6,7]. In 2004, the United Nations Conference on Trade and Development (UNCTAD) proposed the Liner Shipping Connectivity Index (LSCI) for shipping connectivity [8] and later extended the LSCI to the Port-LSCI (PLSCI) in 2019 to quantify the efficiency of a port for handling ships and cargo. Many researchers have, therefore, started to use the PLSCI for port competitiveness assessments. For example, Tovar and Wall [9] used the PLSCI to analyze the relationship between port connectivity and port productivity, and conducted case studies in 16 Spanish ports. Their results illustrated a strong positive correlation between the connectivity and efficiency of ports. In addition to the PLSCI, researchers have developed a variety of port performance indicators [10]. However, these indicators tend to focus on the capabilities of ports themselves with little regard for the global characteristics and the directionality of shipping networks, which are important to maritime transport.

With the development of network science, the quantitative analysis of the topological characteristics of liner shipping networks (LSNs), globally or within a specific area, is a relatively new research topic [11,12]. Space-L and Space-P theories are used to represent the topology of complex maritime transport networks [13]. In Space-L, a link is created between consecutive stops in one route; in Space-P, all ports that belong to the same route are connected. Both methods have been adopted in shipping network research [14,15]. As routes from/to a port are often different, a directed LSN using Space-L reflects the practical situation more faithfully [16,17,18,19].

The rapid increase in world trade and maritime transportation has increased competition among ports. The performance of ports has been investigated by many researchers using competitive network theory [20]. Jiang, et al. [21] defined the variation in the weighted average shortest path length of the liner shipping network when trans-shipment is enabled or not as port connectivity, and conducted a case study for major ports in the Asia–Pacific region. Tovar et al. [22] believed that indicators such as the degree and betweenness from the perspective of the network structure and the freight volume from the perspective of the port itself may characterize the connectivity and performance of ports. However, these approaches do not comprehensively characterize the accessibility of the transport system [23,24] and there is a lack of analysis and a lack of an application to network directionality.

Accessibility has been a research hotspot in transport geography and other research areas since its inception in 1959 [25,26]. Shipping accessibility underpins maritime network development, port planning, and economic benefits to regional and national economies [27,28].

Within a network, the accessibility of a node can be calculated from two aspects: the transport capability of the node relative to the overall network and the attractiveness of the node. The transport capability of the overall network depends on the links connected to the node and transit impedance among the links; in other words, the transport topological impedance structure of the network. The attractiveness of a node depends on the node itself; for example, the size/capacity and efficiency of the node in dealing with cargo and ship operations. Both aspects should be considered in accessibility evaluations for ports in an LSN; previous studies have rarely addressed them.

In 2020, the COVID-19 epidemic caused global health and economic crises that disrupted shipping and trade patterns and severely affected global growth prospects [29]. The UNCTAD predicted that the volume of international maritime trade would drop by 4.1% in 2020 [30]. Researchers found that during the first half of 2020 there were decreases in maritime traffic of 70.2% in exclusive economic zones [31]. The evaluation of the accessibility of ports from the perspective of the overall LSN and the port itself could be helpful for a better understanding of the global trade flow and structure in the post-COVID-19 pandemic era.

In the present study, we constructed a directed GLSN using Space-L with the latest routes (2021) collected from the websites of the top six liner shipping companies. An accessibility evaluation model using the PLSCI and betweenness of the ports was proposed. The topological and community characteristics of the GLSN were analyzed and interpreted to better understand the global liner shipping network and the accessibility of ports in 2021.

The main contributions of this paper are: (1) the liner shipping data collected in 2021 could improve the understanding of the status of global maritime transport; (2) the topological features and community structure of the GLSN in the post-COVID-19 era were analyzed; and (3) a comprehensive accessibility model considering network directionality was proposed and applied to the GLSN.

## 2. Materials and Methods

### 2.1. Data

The data used in this article included the service routes of the liner companies, the PLSCI, and the basic attributes of the port such as the country code, port latitude, and port longitude, as shown in Table 1.

#### 2.1.1. Shipping Lines

**Figure 1:**
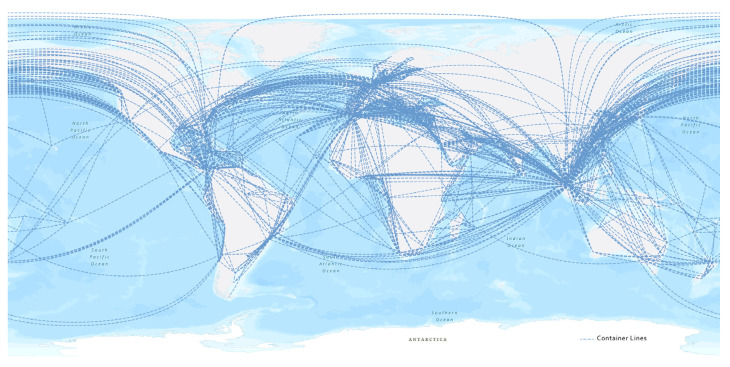
Route linkages (not actual routes) of the 6 liner shipping operators showing interconnections between ports (nodes). We added the attribute of transit time to the linkages so that linkages could reflect the route of real maritime transport.

We collected the service schedules published on the websites of the top six liner shipping operators (about 71.3% of the global TEUs in 2021, according to Alphaliner.com, as shown in Table 2) from 13 July to 26 July 2021. The route data included the departure port, arrival port, and transit time between ports. The basic attributes of the ports (e.g., the coordinates and country code), bought from IHS Markit, were matched with the name and location of the ports. The GLSN included 564 unique ports (nodes) and 9474 routes (repeated routes of different companies were merged into 2971 directed links), as shown in Figure 1.

#### 2.1.2. PLSCI

The Liner Shipping Connectivity Index (LSCI), published by the UNCTAD annually since 2004, evaluates the degree of integration for countries connected to the global liner shipping network. It was further improved by the Port-LSCI (PLSCI), which covers more than 900 container ports around the world and is updated quarterly. We collected the PLSCI data from GLSN ports in the second quarter of 2021 from the UNCTAD website.

### 2.2. Methods

The following methods were applied to determine the network linkages and topology of the port connections in 2021. The complex network analysis methods used included the average degree, average clustering coefficient, average shortest path length, and betweenness; these were applied to the GLSN. The Leiden community detection algorithm was used to discover the trade topology structure of the GLSN and the modularity of the partition result was calculated. Finally, an accessibility evaluation model was proposed.

#### 2.2.1. Complex Network Analysis Factors

In this section, definitions are provided for the factors used in the GLSN, including the average degree, average clustering coefficient, average shortest path length, and betweenness.

**Degree and average degree:** The degree of a node is the number of links adjacent to the node. The in-degree and out-degree are considered separately in directed networks. The average degree 〈k〉 for a directed network is defined as follows:

(1) $$\langle k\rangle =\frac{1}{N}\overset{N}{\underset{i=1}{\sum }}k_{i}^{in}+\frac{1}{N}\overset{N}{\underset{i=1}{\sum }}k_{i}^{out}=\frac{L}{N}$$

where N represents the total number of nodes in the network; $k_{i}^{in}$ represents the number of links that point into node i; $k_{i}^{out}$ represents the number of links that point out from node i to other nodes; and L represents the total number of links (regardless of direction) in the network.

**Average shortest path length:** The average shortest path length (ASPL) is defined as the average number of steps along the shortest paths for all possible pairs of network nodes. It is a measure of the efficiency of information or mass transport on a network.

Consider a network G with the set of vertices V; $dist\left(v_{1},v_{2}\right)$ denotes the shortest path between $v_{1}$ and $v_{2}\left(v_{1},v_{2}\in V\right)$. If $dist\left(v_{1},v_{2}\right)=0$, then $has\_path\left(v_{1},v_{2}\right)=0$ if there is no path between $v_{1}$ and $v_{2}$ or $v_{1}=v_{2}$. However, if $has\_path\left(v_{1},v_{2}\right)=1$ then there is a path from $v_{1}$ to $v_{2}$. The ASPL for network G can then be defined as:

(2) $$ASPL_{G}=\frac{\sum_{i,j}^{N}dist\left(v_{i},v_{j}\right)}{\sum_{i,j}^{N}has\_path\left(v_{1},v_{2}\right)}$$

where, $\overset{N}{\underset{i,j}{\sum }}dist\left(v_{i},v_{j}\right)$ represents the sum of all shortest path lengths and $\overset{N}{\underset{i,j}{\sum }}has\_path\left(v_{1},v_{2}\right)$ represents the total number of paths [32].

**Average clustering coefficient:** The degree of clustering of a whole network is captured by the average clustering coefficient C, representing the average of $c_{v}$ over all nodes in the network:

(3) $$C=\frac{1}{N}\underset{v\in G}{\sum }c_{v}$$

The clustering coefficient for each node in the directed network is calculated as follows, according to [18]:

(4) $$c_{v}=\frac{2T_{v}}{k_{v}^{tot}\left(k_{v}^{tot}-1\right)-2k_{v}^{↔}}$$

where $k_{v}^{tot}$ is the sum of the in-degree and out-degree of the node v in the network, $T_{v}$ is the number of directed triangles passing through node v, and $k_{v}^{↔}$ is the reciprocal degree of v.

**Betweenness centrality:** Betweenness centrality is an indicator measuring the influence of the nodes based on the shortest path. The betweenness centrality of node v is the sum of the fraction of the shortest paths of all pairs that pass through v:

(5) $$C_{B}\left(v\right)=\underset{s,t\in V}{\sum }\frac{\sigma \left(s,t|v\right)}{\sigma \left(s,t\right)}$$

where σ(s,t) is the number of shortest paths between node s and node t and σ(s,t|v) is the number of those paths passing through a node v, other than s or t. If s=t, σ(s,t); if v∈s,t, σ(s,t)=0.

Betweenness centrality can be normalized for directed networks as:

(6) $$C_{B}{\left(v\right)}_{norm}=\frac{C_{B}\left(v\right)}{\left(N-1\right)\left(N-2\right)}$$

**Community detection:** The Leiden algorithm package for Python [33] was used for the shipping community detection. The Leiden algorithm [34], which extends the Louvain algorithm [35], is widely regarded as one of the best algorithms for detecting communities. The frequency of occurrence for each route can be used as the weight of the route whilst detecting the communities.

**Modularity:** Modularity is a measurement for the partitioning of the network into communities. A higher modularity value indicates a stronger internal connection or cohesiveness within a community. In practice, a value between 0.3~0.7 is considered to be a good indicator of a significant cohesive community structure in networks [35,36,37]. Modularity can be expressed as:

(7) $$Q=\frac{1}{2L}\underset{i,j}{\sum }\left(A_{ij}-\frac{k_{i}k_{j}}{2L}\right)\sigma \left(c_{i},c_{j}\right)$$

where A is the adjacency matrix for the network and $k_{i}$ is the total degree of node i. Specifically, if i and j are in the same community, then $\sigma \left(c_{i},c_{j}\right)=1$; otherwise, it is 0.

#### 2.2.2. Accessibility Evaluation Model

Combining the PLSCI, betweenness centrality for ports, and transit time between ports, the accessibility for port liner shipping transportation is defined as:

(8) $$A_{from\_i}=\overset{m}{\underset{j=1}{\sum }}\frac{\sqrt{B_{j}C_{j}O_{ij}}}{T_{ij}}$$

(9) $$A_{to\_i}=\overset{n}{\underset{j=1}{\sum }}\frac{\sqrt{B_{i}C_{i}O_{ji}}}{T_{ji}}$$

where $A_{from\_i}$ and $A_{to\_i}$ are the accessibility from/to port i, respectively; m and n are the total number of routes from/to port i, respectively; $T_{ij}$ and $T_{ji}$ are the transit times from port i/j to port j/i, respectively; $C_{i}$ and $C_{j}$ are the PLSCIs of port i/j, respectively; $B_{i}$ and $B_{j}$ are the betweenness centralities of port i/j, respectively; and $O_{ij}$ and $O_{ji}$ are the occurrence times of link ij/ji, respectively.

## 3. Results

**Figure 2:**
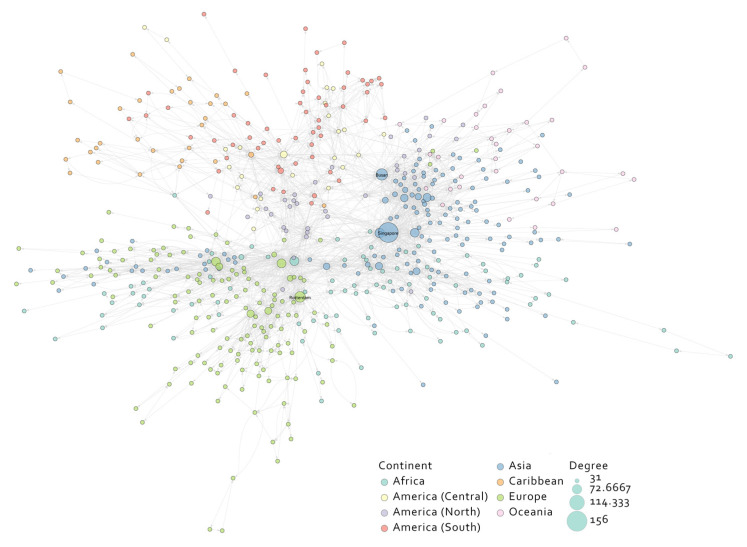
The GLSN. The size of a node is based on its degree; we set the size of nodes with a degree between 1 and 31 as the minimum and the size of nodes with a degree of 156 (Singapore) as the maximum. The color of the nodes in the diagram varies depending on the continent to which they belong. The continent classification is provided by IHS Markit.

Based on the collected data, we constructed a directed GLSN, as shown in Figure 2. The route attributes included the average transit time between nodes and the frequency of route appearances. The port attributes included the country that the port belonged to and the corresponding PLSCI of the port.

### 3.1. Topological Characteristics of the GLSN

**Figure 3:**
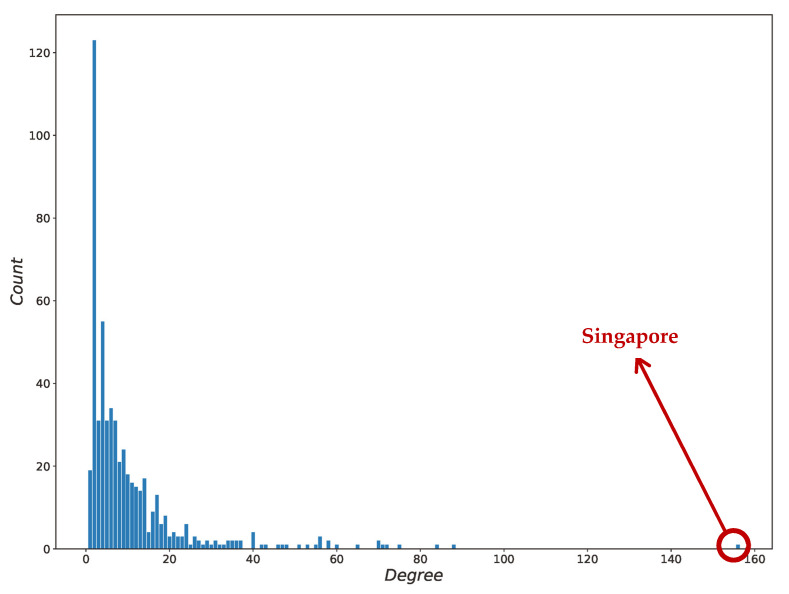
Degree distribution of GLSN.

The average degree of the GLSN was 5.27, the average clustering coefficient was 0.33, and the average shortest path length was 4.10. The degree distribution of the GLSN, as shown in Figure 3, demonstrated that most ports had few shipping routes. However, there were several important ports such as Singapore port that had a considerable number of routes from/to different ports. A relatively high average degree and a small average shortest path length indicated that the GLSN conformed to the characteristics of a small-world network, which was consistent with the findings of previous works [13,38,39].

**Figure 4:**
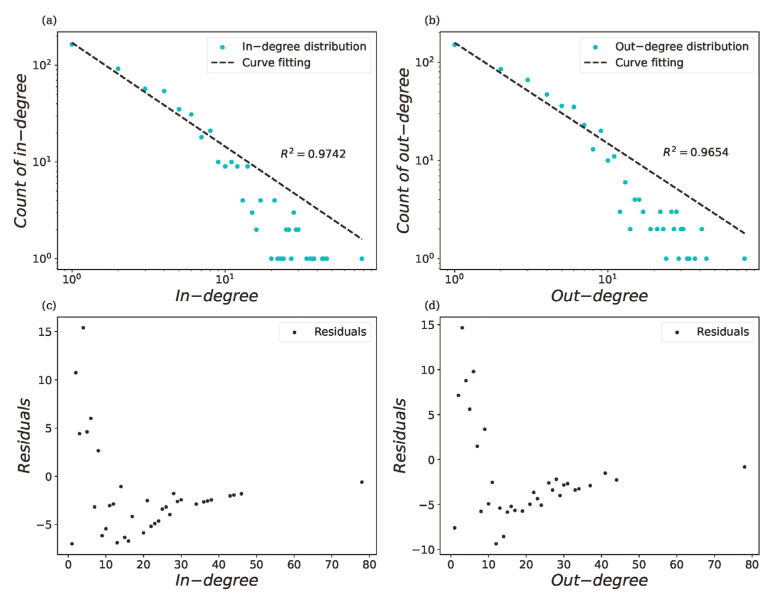
In-degree and out-degree power-law curve fitting. (**a**,**b**) illustrated the power-law curve fitting results for in-degree and out-degree of GLSN respectively. (**c**,**d**), illustrated their fitting residuals.

Previous studies [19,40,41,42] concluded that the GLSN should be a scale-free network and that the in-degree and out-degree of a directed network should conform to the power-law distribution, respectively [43]. We tested the power-law fitting for the in-degree and out-degree distribution of the GLSN in log-scaled axes (Figure 4a,b); both R-square values were larger than 0.96. It can be seen from Figure 4c,d that the residuals of the power-law distribution fitting were higher at the small-degree nodes, but became smaller as the degree increased. The fitting result showed that the GLSN was a scale-free network for the in-degree and out-degree. That is to say, a few nodes in the GLSN had a greater number of links and these nodes were called hubs. Hubs typically had links from the entire network serving as links between different parts of the network, thus showing a small-world property. For example, the Singapore port, mentioned above, had the highest degree (including the out-degree and in-degree, respectively); it is an important hub port for East Asian and European trade.

Singapore or other hub ports are transit points for world maritime trade where goods are distributed. The in-degree and out-degree of most hub ports are similar such as Singapore (in-degree 78; out-degree 78), Busan (46, 44), and Rotterdam (43, 41). However, several ports also have large differences in their in-degree and out-degree. As can be seen from Table 3, hub ports such as Tanger Med and Algeciras have an obvious difference in their in-degree and out-degree. The same situation occurs in other ports such as Sydney, Veracruz, and Tianjin.

**Figure 5:**
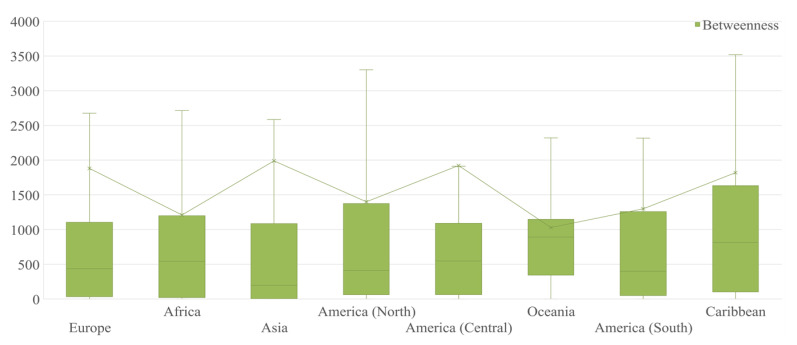
Box plot for betweenness of ports in different continents. Values within the box lie between the inter-quartile range of 0.25 to 0.75. The bar within the box represents the median value and the bar outside the box represents the extreme outlier range.

The ranking for the betweenness of ports in the GLSN is partly shown in Table 4. Singapore port had the highest betweenness centrality, reaching 0.26 after normalization, followed by Rotterdam port at 0.13 and Busan port at 0.11. The ports with a high betweenness centrality belonged to a wide range of countries, but they were mainly distributed in Asia and Europe. As seen in Figure 5, Asian and European ports had a higher average betweenness than the other continents. However, the difference of betweenness among continents was not highly significant.

**Figure 6:**
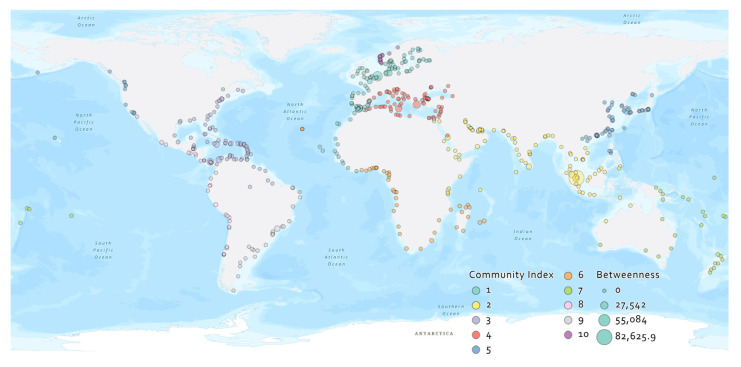
Communities detected in GLSN. Nodes are represented in their real coordinates (7 out of 564 ports that were not strongly connected to the major component of the GLSN are excluded).

The Leiden algorithm was used to determine the community division using three million randomly simulated divisions. Modularity was used to evaluate the result of each division. The partitioning based on the 3 million divisions divided the 557 ports into 10 communities, as shown in Figure 6. The largest modularity value observed was 0.6433.

Figure 6 shows the global spatial distribution of the ports belonging to each of the 10 communities described in Table 5 as communities C1 to C10. The number of ports in the top 6 communities accounted for 82.45% of the total number of ports. C5 had the highest average degree at 6.00, the second highest average clustering coefficient at 0.44, and the third shortest path at 2.54; it performed the best in the 3 indicators among the top 6 biggest communities.

### 3.2. Accessibility of Ports in the GLSN

We obtained the PLSCI of global ports and matched the PLSCI in Q2 of 2021 (consistent with the period of obtaining the route data) with the ports in the GLSN for the accessibility calculations. Table 6 shows the top 30 ports with the highest PLSCI.

**Figure 7:**
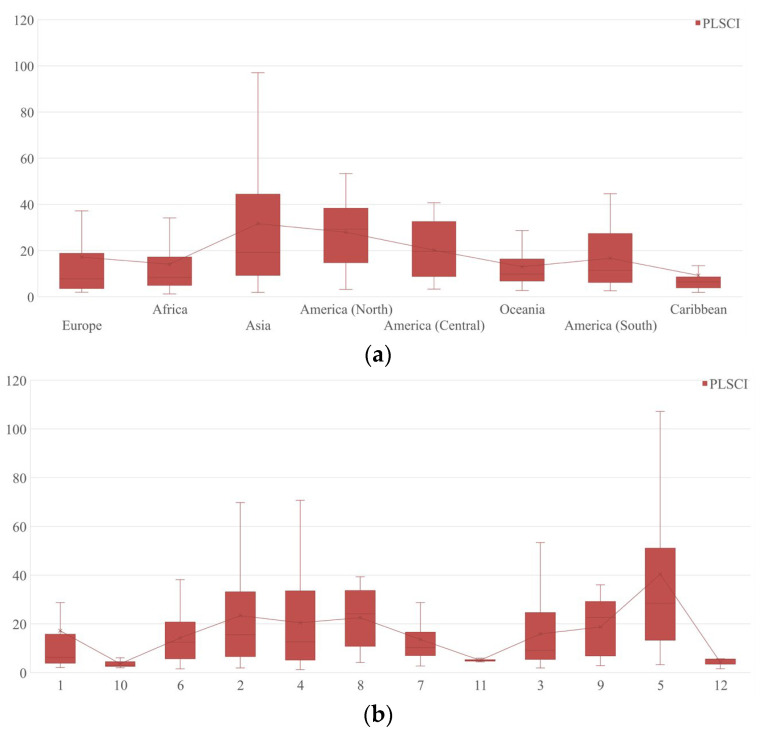
Box plots for PLSCI of ports in GLSN. Several ports (20) that were not matched to a PLSCI are excluded from this figure. (**a**) Box plot for PLSCI of ports in different continents; (**b**) Box plot for PLSCI of ports in different communities.

Almost all of the top 30 ports with the highest PLSCI were European and Asian ports, and 11 of the ports were in China (including Hong Kong, Macao, and Taiwan). As for the PLSCI of all ports, Figure 7a shows that the North American ports had the second highest average as well as the highest median PLSCI. The PLSCI of the Asian ports varied from 1.88 (Ajman port) to 145.85 (Shanghai port). Figure 7b shows that C5, which was mainly located in east Asia and the west coast of North America, had the highest average and median PLSCI.

The accessibility evaluation model was applied to calculate the inbound and outbound accessibility of ports in the GLSN; the top 30 ports are shown in Table 7. Singapore port had the highest inbound, outbound, and total accessibility amongst all ports in the GLSN. This was followed by Port Klang and Rotterdam. Tanjung Pelepas, Busan, and Shanghai port ranked 4–6 in total accessibility. In addition, the 30 ports all belonged to the first to fifth communities; the fifth community, which was mainly located in East Asia, had 11 ports in the ranking list.

## 4. Discussion

We constructed a directed GLSN and found that its in-degree and out-degree conformed to a power-law distribution, implying that a small number of hub nodes had a large number of links. Many studies consider the liner shipping network to be an undirected network, ignoring the directionality of the transportation flow. As shown in Table 3, Tanger Med port had a node degree of 72; its in-degree was 16 higher than its out-degree. Qingdao port had a node degree of 40, but its in-degree was 16 higher than its out-degree. These ports may be gateway ports that import goods into the hinterland. On the other hand, ports such as Algeciras and Le Havre, whose out-degrees were higher than their in-degrees, may be hub ports that export goods to all over the world. In addition, ports with a higher inbound accessibility have advantages of transit distance (time), port location, and port attractiveness, but they are at a higher risk of invasive species.

According to the annual review for maritime transport of UNCTAD, all containerized east–west trade routes among Asia, the Mediterranean, Europe, and North America account for 52.6% of the total freight volume of the world [44]. The community division result showed that the spatial distribution of the main communities conformed to the actual situation of the major routes of container transportation (the number of ports from community 1–5 mainly distributed in Asia, the Mediterranean, Europe, and North America accounted for 59.93% of the total ports in the GLSN). In addition, container port throughput in Asia and Europe accounted for 79.71% of the global throughput, according to the UNCTAD [44]; the same trend emerged in the port accessibility assessment results. Most of the leading 30 ports with the highest accessibility were Asian (17) and European (10) ports.

Regarding port accessibility, although the average PLSCI of the North American ports ranked second (behind Asia), there was no North American port in the total accessibility rank. The average betweenness centrality of the North American ports was relatively low and the transit times of both the trans-pacific routes and the North American–Europe routes were longer than the other major routes. This could be the reason why the overall accessibility of the North American ports was not high.

Regarding port management, an assessment of port accessibility can clarify the state of a port. Hub ports such as Singapore and Hong Kong should maintain their inbound and outbound accessibility at a similar level. Ports with a higher inbound or outbound accessibility such as Hamburg and Nansha should further develop their strengths and enhance their connectivity with the shipping network. For shipping companies, the accessibility of ports could also be useful when designing new routes. For example, a similar level of outbound and inbound accessibility for all ports in a route may reduce blank sailings and improve the efficiency of ships.

Although the data obtained were based on only six main liner companies, they were enough to illustrate the GLSN; the research result could easily be extended to a more detailed dataset. The transit time of the routes was the average value collected from the websites of the liner companies rather than the actual transit times, which may have had a slight impact on the calculation of accessibility. In addition, the data did not contain information such as the ship tonnage or ship carry capacity; therefore, the trade volume for routes was not considered in this study. Despite a few limitations, the GLSN developed to quantitatively analyze the inbound and outbound accessibility of global container ports could be used for subsequent studies.

## 5. Conclusions

The topological characteristics of the GLSN using Space-L from liner shipping companies provided a scale-free network, which indicated that few ports accommodated the majority of links. Community divisions into 10 clusters showed an obvious correspondence with the actual trade flow. The directed accessibility between the inbound and outbound trade flows significantly affected the topological structure. The accessibility evaluation result showed that the Asian ports had the highest total accessibility, with the inbound accessibility close to that of the outbound. The European ports ranked behind the Asian ports. The ports in North America had a relatively low accessibility because of the long transit time and low betweenness. Our research has enhanced the understanding of maritime networks and could provide insights into route optimization as well as other studies such as species invasion and port planning.

In the future, the research in this paper can be expanded in several ways. First, due to the availability of data, our analysis focused on the topographic characteristics of the GLSN. However, other indicators such as port throughput and port efficiency are worth studying. Second, the liner shipping data collected in 2021 reflected the shipping patterns in the post-COVID-19 era. However, the outbreak of war between Russia and Ukraine in 2022 has led to further changes in the patterns of global energy and food trade. It is possible to construct an updated long-term shipping network to analyze the impact of major international incidents such as COVID-19 or regional wars on maritime transport.

## Figures and Tables

**Table 1:** Attributes merged and used in this study.

Attribute Name	Description
Port Name	Port names matched among routes from 6 liner shipping companies, PLSCI data from the UNCTAD, and port data from the IHS market
PLSCI	Published by the UNCTAD quarterly

**Table 2:** Top 6 liner shipping operators ranked by share of TEU (twenty-foot equivalent unit of containers).

Rank ^1^	Operator	TEU	Share	Total Share
1	Maersk	4,221,901	17.1%	17.1%
2	MSC	4,083,743	16.5%	33.6%
3	CMA CGM	2,996,919	12.1%	45.7%
4	COSCO	2,982,192	12.0%	57.7%
5	Hapag-Lloyd	1,782,858	7.2%	64.9%
6	ONE	1,592,173	6.4%	71.3%

^1^ The ranks are from https://alphaliner.axsmarine.com/PublicTop100/index.php, updated on 18 August 2021.

**Table 3:** Variation between the in-degree and out-degree of ports.

Port	Total Degree	In-Degree	Out-Degree	Variation
Tanger Med	72	44	28	16
Qingdao	40	28	12	16
New York	34	23	11	12
Sydney	14	11	3	8
Veracruz	16	12	4	8
Tianjin	20	14	6	8
Wellington	18	5	13	−8
Algeciras	73	31	42	−11
Le Havre	40	14	26	−12
Charleston	35	11	24	−13

**Table 4:** Top 30 ports with the highest betweenness centrality.

Rank	Port	Country	Continent	Betweenness	Normalized Betweenness
1	Singapore	Singapore	Asia	82,327	0.26
2	Rotterdam	Netherlands	Europe	42,172	0.13
3	Busan	South Korea	Asia	35,468	0.11
4	Algeciras	Spain	Europe	28,962	0.09
5	Tanger Med	Morocco	Africa	28,557	0.09
6	Piraeus	Greece	Europe	28,445	0.09
7	Manzanillo (Panama)	Panama	America (Central)	22,414	0.07
8	Marsaxlokk	Malta	Europe	21,712	0.07
9	Tanjung Pelepas	Malaysia	Asia	21,057	0.07
10	Port Klang	Malaysia	Asia	18,436	0.06
11	Cartagena (Colombia)	Colombia	America (South)	18,174	0.06
12	Kingston (Jamaica)	Jamaica	Caribbean	17,678	0.06
13	Bremerhaven	Germany	Europe	16,618	0.05
14	Santos	Brazil	America (South)	15,541	0.05
15	Hamburg	Germany	Europe	15,521	0.05
16	Jebel Ali	United Arab Emirates	Asia	13,263	0.04
17	Colombo	Sri Lanka	Asia	12,323	0.04
18	Jeddah	Saudi Arabia	Asia	10,782	0.03
19	Le Havre	France	Europe	10,276	0.03
20	Caucedo	Dominican Republic	Caribbean	8753	0.03
21	Antwerp	Belgium	Europe	8362	0.03
22	Valencia	Spain	Europe	7670	0.02
23	Shanghai	People’s Republic of China	Asia	7486	0.02
24	Yantian	People’s Republic of China	Asia	7076	0.02
25	New York	United States of America	America (North)	7011	0.02
26	Balboa	Panama	America (Central)	6760	0.02
27	Auckland	New Zealand	Oceania	6717	0.02
28	Hong Kong	Hong Kong, China	Asia	6508	0.02
29	Port Newark	United States of America	America (North)	6379	0.02
30	Salalah	Oman	Asia	6304	0.02

America (Central), including Mexico, Panama, and Belize, of the ports of 8 countries according to the port data of IHS Markit.

**Table 5:** Description and indicators of the 10 communities determined from the GLSN analysis.

ID	Description	Indicators
**C1**	The largest community was mainly distributed in **Europe and north-west Africa** and included 105 ports from 31 countries	Greatest betweenness centrality ports included Rotterdam, Algeciras, and Tanger Med. The average degree in this community was 4.24, average clustering coefficient was 0.34, and average shortest path length was 3.12
**C2**	The second community was mainly located in **south-east Asia, west Asia, and north-east Africa** and included 88 ports from 33 countries	Greatest betweenness centrality ports included Singapore, Tanjung Pelepas, and Jebel Ali. The average degree in this community was 4.32, average clustering coefficient was 0.44, and average shortest path length was 2.83
**C3**	The third community was mainly located on the **east coast of North America and Central America** and consisted of 87 ports from 39 countries	Major ports included Manzanillo (Panama), Cartagena (Colombia), and New York. The average degree in this community was 3.49, average clustering coefficient was 0.31, and average shortest path length was 3.44
**C4**	The fourth community was scattered around the **Mediterranean Sea** and consisted of 80 ports from 24 countries	Major ports included Piraeus, Marsaxlokk, and Valencia. The average degree in this community was 4.94, average clustering coefficient was 0.31, and average shortest path length was 2.89
**C5**	Ports of the fifth community were mainly distributed in **east Asia and the west coast of North America** and consisted of 65 ports from 8 countries	Major ports included Busan, Shanghai, Hong Kong, and Los Angeles. The average degree in this community was 6.00, average clustering coefficient was 0.44, and average shortest path length was 2.54
**C6**	Ports of the sixth community were mainly scattered around the **south coast of Africa** and consisted of 40 ports from 18 countries	Major ports included Durban and Pointe Noire. The average degree in this community was 3.00, average clustering coefficient was 0.33, and average shortest path length was 3.68
**C7**	The seventh community consisted of 30 ports from 12 countries from **Oceania**	Major ports included Auckland and Brisbane. The average degree in this community was 2.57, average clustering coefficient was 0.30, and average shortest path length was 3.73
**C8**	The eighth community consisted of 27 ports from 11 countries from the **west coast of South America and Central America**	Major ports included Balboa and Callao. The average degree in this community was 4.37, average clustering coefficient was 0.47, and average shortest path length was 2.34
**C9**	The ninth community consisted of 22 ports from 3 countries distributed on the **east coast of South America**	Major ports included Santos and Paranagua. The average degree in this community was 3.64, average clustering coefficient was 0.36, and average shortest path length was 2.30
**C10**	The tenth community consisted of 13 ports from **Norway**	Major ports included Haugesund and Aalesund. The average degree in this community was 1.69, average clustering coefficient was 0.29, and average shortest path length was 2.88

**Table 6:** Top 30 ports with the highest PLSCI.

Rank	Port	Country	Continent	PLSCI
1	Shanghai	People’s Republic of China	Asia	145.85
2	Singapore	Singapore	Asia	128.52
3	Ningbo-Zhoushan	People’s Republic of China	Asia	125.73
4	Busan	South Korea	Asia	119.15
5	Hong Kong	Hong Kong, China	Asia	107.16
6	Qingdao	People’s Republic of China	Asia	97.03
7	Rotterdam	Netherlands	Europe	95.67
8	Port Klang	Malaysia	Asia	93.34
9	Antwerp	Belgium	Europe	93.21
10	Kaohsiung	Taiwan, China	Asia	88.52
11	Shekou-Chiwan	People’s Republic of China	Asia	85.66
12	Xiamen	People’s Republic of China	Asia	85.57
13	Yantian	People’s Republic of China	Asia	85.13
14	Nansha	People’s Republic of China	Asia	81.17
15	Hamburg	Germany	Europe	80.87
16	Jebel Ali	United Arab Emirates	Asia	78.12
17	Tianjin	People’s Republic of China	Asia	77.54
18	Colombo	Sri Lanka	Asia	74.90
19	Valencia	Spain	Europe	70.70
20	Tanjung Pelepas	Malaysia	Asia	69.78
21	Algeciras	Spain	Europe	69.51
22	Le Havre	France	Europe	67.88
23	Tanger Med	Morocco	Africa	67.35
24	Laem Chabang	Thailand	Asia	67.27
25	Bremerhaven	Germany	Europe	65.51
26	Barcelona	Spain	Europe	65.05
27	Dalian	People’s Republic of China	Asia	63.79
28	Gwangyang	South Korea	Asia	62.32
29	Piraeus	Greece	Europe	62.28
30	Yokohama	Japan	Asia	60.52

**Table 7:** Top 30 ports with the highest total accessibility and their community index.

Rank	Port	Community Number	Outbound Accessibility	Inbound Accessibility	Total Accessibility
1	Singapore	2	195.67	194.69	390.36
2	Port Klang	2	82.58	97.30	179.88
3	Rotterdam	1	80.25	86.82	167.07
4	Tanjung Pelepas	2	78.35	57.94	136.29
5	Busan	5	65.98	64.88	130.86
6	Shanghai	5	60.79	61.71	122.50
7	Hong Kong	5	45.52	44.60	90.12
8	Ningbo	5	39.62	47.83	87.45
9	Algeciras	1	41.83	44.87	86.71
10	Hamburg	1	35.36	51.07	86.42
11	Tanger Med	1	45.39	34.01	79.39
12	Antwerp	1	42.96	33.35	76.30
13	Bremerhaven	1	34.97	33.76	68.73
14	Yantian	5	35.15	33.34	68.50
15	Shekou	5	21.91	20.54	42.45
16	Le Havre	1	21.94	20.09	42.03
17	Piraeus	4	19.72	19.90	39.62
18	Kaohsiung	5	18.42	20.05	38.47
19	Colombo	2	17.08	17.57	34.64
20	Nansha	5	16.97	13.56	30.53
21	Cartagena (Colombia)	3	15.85	14.07	29.92
22	London Gateway	1	13.33	14.16	27.49
23	Manzanillo (Panama)	3	13.77	12.81	26.59
24	Qingdao	5	14.03	11.69	25.72
25	Marsaxlokk	4	9.58	15.66	25.24
26	Xiamen	5	11.95	12.81	24.76
27	Valencia	4	10.72	12.65	23.37
28	Jebel Ali	2	10.50	12.74	23.24
29	Jeddah	2	11.44	10.65	22.09
30	Yokohama	5	9.16	7.99	17.15

## Data Availability

The PLSCI used in this study is a publicly available dataset from the UNCTAD and it can be found here: [https://unctadstat.unctad.org] (accessed on 11 July 2022).

## References

[1] UNCTAD (2017). Review of Maritime Transport 2017.

[2] Lin P.-C., Kuo S.-Y., Chang J.-H. (2020). The direct and spillover effects of liner shipping connectivity on merchandise trade. Marit. Bus. Rev..

[3] Pan J.-J., Bell M.G.H., Cheung K.-F., Perera S., Yu H. (2019). Connectivity analysis of the global shipping network by eigenvalue decomposition. Marit. Policy Manag..

[4] Ducruet C. (2020). The geography of maritime networks: A critical review. J. Transp. Geogr..

[5] Valentine V.F., Benamara H., Hoffmann J. (2013). Maritime transport and international seaborne trade. Marit. Policy Manag..

[6] Yin J., Shi J. (2018). Seasonality patterns in the container shipping freight rate market. Marit. Policy Manag..

[7] Hoffmann J., Saeed N., Sødal S. (2019). Liner shipping bilateral connectivity and its impact on South Africa’s bilateral trade flows. Marit. Econ. Logist..

[8] Mishra V.K., Dutta B., Goh M., Figueira J.R., Greco S. (2021). A robust ranking of maritime connectivity: Revisiting UNCTAD’s liner shipping connectivity index (LSCI). Marit. Econ. Logist..

[9] Tovar B., Wall A. (2021). The relationship between port-level maritime connectivity and efficiency. J. Transp. Geogr..

[10] Talley W.K., Ng M., Marsillac E. (2014). Port service chains and port performance evaluation. Transp. Res. Part E Logist. Transp. Rev..

[11] Kosowska-Stamirowska Z. (2020). Network effects govern the evolution of maritime trade. Proc. Natl. Acad. Sci. USA.

[12] Wan C., Zhao Y., Zhang D., Yip T.L. (2021). Identifying important ports in maritime container shipping networks along the Maritime Silk Road. Ocean Coast. Manag..

[13] Hu Y., Zhu D. (2009). Empirical analysis of the worldwide maritime transportation network. Phys. A Stat. Mech. Its Appl..

[14] Xu M., Pan Q., Muscoloni A., Xia H., Cannistraci C.V. (2020). Modular gateway-ness connectivity and structural core organization in maritime network science. Nat. Commun..

[15] Cheung K.-F., Bell M.G., Pan J.-J., Perera S. (2020). An eigenvector centrality analysis of world container shipping network connectivity. Transp. Res. Part E Logist. Transp. Rev..

[16] Garlaschelli D., Loffredo M.I. (2004). Fitness-Dependent Topological Properties of the World Trade Web. Phys. Rev. Lett..

[17] Garlaschelli D., Loffredo M.I. (2005). Structure and evolution of the world trade network. Phys. A Stat. Mech. Its Appl..

[18] Fagiolo G. (2007). Clustering in complex directed networks. Phys. Rev. E.

[19] Calatayud A., Mangan J., Palacin R. (2017). Vulnerability of international freight flows to shipping network disruptions: A multiplex network perspective. Transp. Res. Part E Logist. Transp. Rev..

[20] Serrano M., Boguñá M. (2003). Topology of the world trade web. Phys. Rev. E.

[21] Jiang J., Lee L.H., Chew E.P., Gan C.C. (2015). Port connectivity study: An analysis framework from a global container liner shipping network perspective. Transp. Res. Part E Logist. Transp. Rev..

[22] Tovar B., Hernández R., Rodriguez-Deniz H. (2015). Container port competitiveness and connectivity: The Canary Islands main ports case. Transp. Policy.

[23] Luo D., Cats O., van Lint H., Currie G. (2019). Integrating network science and public transport accessibility analysis for comparative assessment. J. Transp. Geogr..

[24] Wang P., Hu Q., Xu Y., Mei Q., Wang F. (2021). Evaluation methods of port dominance: A critical review. Ocean Coast. Manag..

[25] Hansen W.G. (1959). How accessibility shapes land use. J. Am. Inst. Plan..

[26] van Wee B. (2016). Accessible accessibility research challenges. J. Transp. Geogr..

[27] Fugazza M., Hoffmann J. (2017). Liner shipping connectivity as determinant of trade. J. Shipp. Trade.

[28] Xu M., Pan Q., Xia H., Masuda N. (2020). Estimating international trade status of countries from global liner shipping networks. R. Soc. Open Sci..

[29] Xu L., Yang S., Chen J., Shi J. (2021). The effect of COVID-19 pandemic on port performance: Evidence from China. Ocean Coast. Manag..

[30] UNCTAD (2020). Review of Maritime Transport 2020.

[31] March D., Metcalfe K., Tintoré J., Godley B.J. (2021). Tracking the global reduction of marine traffic during the COVID-19 pandemic. Nat. Commun..

[32] Mao G., Zhang N. (2013). Analysis of Average Shortest-Path Length of Scale-Free Network. J. Appl. Math..

[33] Traag V.A., Waltman L., Van Eck N.J. (2019). From Louvain to Leiden: Guaranteeing well-connected communities. Sci. Rep..

[34] Blondel V.D., Guillaume J.-L., Lambiotte R., Lefebvre E. (2008). Fast unfolding of communities in large networks. J. Stat. Mech. Theory Exp..

[35] Clauset A., Newman M.E.J., Moore C. (2004). Finding community structure in very large networks. Phys. Rev. E.

[36] Newman M.E.J., Girvan M. (2004). Finding and evaluating community structure in networks. Phys. Rev. E.

[37] Newman M.E.J. (2006). Modularity and community structure in networks. Proc. Natl. Acad. Sci. USA.

[38] Caschili S., Medda F., Parola F., Ferrari C. (2014). An Analysis of Shipping Agreements: The Cooperative Container Network. Networks Spat. Econ..

[39] Liu C., Wang J., Zhang H. (2017). Spatial heterogeneity of ports in the global maritime network detected by weighted ego network analysis. Marit. Policy Manag..

[40] Ducruet C., Lee S.-W., Ng A.K. (2010). Centrality and vulnerability in liner shipping networks: Revisiting the Northeast Asian port hierarchy. Marit. Policy Manag..

[41] Wang N., Wu N., Dong L.-L., Yan H.-K., Wu D. (2016). A study of the temporal robustness of the growing global container-shipping network. Sci. Rep..

[42] Peng P., Cheng S., Chen J., Liao M., Wu L., Liu X., Lu F. (2018). A fine-grained perspective on the robustness of global cargo ship transportation networks. J. Geogr. Sci..

[43] Barabási A.-L. (2016). Network Science.

[44] UNCTAD (2021). Review of Maritime Transport 2021.

